# Cell Inertia: Predicting Cell Distributions in Lung Vasculature to Optimize Re-endothelialization

**DOI:** 10.3389/fbioe.2022.891407

**Published:** 2022-04-27

**Authors:** Jason K.D. Chan, Eric A. Chadwick, Daisuke Taniguchi, Mohammadali Ahmadipour, Takaya Suzuki, David Romero, Cristina Amon, Thomas K. Waddell, Golnaz Karoubi, Aimy Bazylak

**Affiliations:** ^1^ Department of Mechanical and Industrial Engineering, University of Toronto, Toronto, ON, Canada; ^2^ Latner Thoracic Surgery Laboratories, Toronto General Hospital Research Institute, University Health Network, Toronto General Hospital, University of Toronto, Toronto, ON, Canada; ^3^ Institute of Biomedical Engineering (BME), University of Toronto, Toronto, ON, Canada; ^4^ Department of Thoracic Surgery, Institute of Development, Aging and Cancer, Tohoku University, Sendai, Japan; ^5^ Institute of Medical Sciences, University of Toronto, Toronto, ON, Canada; ^6^ Department of Laboratory Medicine and Pathobiology, University of Toronto, Toronto, ON, Canada

**Keywords:** lung tissue engineering, lung regeneration, computational fluid dynamics, cell seeding, re-endothelialization, inertia

## Abstract

We created a transient computational fluid dynamics model featuring a particle deposition probability function that incorporates inertia to quantify the transport and deposition of cells in mouse lung vasculature for the re-endothelialization of the acellular organ. Our novel inertial algorithm demonstrated a 73% reduction in cell seeding efficiency error compared to two established particle deposition algorithms when validated with experiments based on common clinical practices. We enhanced the uniformity of cell distributions in the lung vasculature by increasing the injection flow rate from 3.81 ml/min to 9.40 ml/min. As a result, the cell seeding efficiency increased in both the numerical and experimental results by 42 and 66%, respectively.

## Introduction

Approximately 3.9 million people die from end-stage lung disease annually worldwide (Labaki and Han, 2020). Lung transplantation remains the only definitive treatment for end-stage lung disease even though only a small portion of patients may be eligible (Nathan, 2015). Furthermore, many complications arise due to the immunological response to donor organs, leading to a median survival rate of only 5.8 years post-transplantation (Thabut and Mal, 2017). Decellularization and recellularization of whole lungs is a nascent lung bioengineering approach for reducing graft rejection following transplantation (Ott et al., 2010; Petersen et al., 2010). In this procedure, cells are removed from the donor organ via a process known as decellularization, which leaves behind an extracellular matrix rich lung scaffold with intact vessel and airway architecture. The organ is then repopulated with recipient-specific cells in a process called recellularization to reduce the likelihood of an autoimmune response, thereby reducing the rate of organ rejection. While creation of a recellularized functional bioartificial lung remains a long-term objective, there has been significant focus on decellularization and recellularization approaches to achieve the first step of fully repopulated scaffolds. The overall process still requires optimization with recellularization being especially challenging (Prakash et al., 2015; Uriarte et al., 2018). This challenge is exacerbated by that fact that to date, defining and optimizing parameters for decellularization and recellularization have relied upon experimental trial-and-error investigations which involve high costs and significant time and effort (Wendt et al., 2009).

Successful decellularization of the lung, which preserves the 3D organ structure while removing cells, must be established as the first step to achieve successful recellularization (Tsuchiya et al., 2014). Over the past decade, there have been significant advancements in the optimization of decellularization for lung scaffolds resulting in a several well validated detergent-based protocols in murine and porcine animal models (Gilpin et al., 2014; Tebyanian et al., 2017; Uhl et al., 2017; Urbano et al., 2017; Ohata and Ott, 2020). Recellularization on the other hand remains the biggest challenge in producing viable bioartificial lungs (Ohata and Ott, 2020). For the lung, recellularization, at a minimum, encompasses epithelialization and endothelialization. For both processes, specific cell populations and cell types must be deposited at appropriate sites on the scaffolds. One critical consideration is that cell seeding efficiency (percentage of total cells that are deposited) should be maximized to avoid the waste of donor cells, which can be costly and time consuming to attain (Olivares and Lacroix, 2012; Marín et al., 2018). Recellularization of lung scaffolds with pluripotent-derived cell sources, for example, can take over 60 days of culture *in vitro* prior to delivery into the acellular lung scaffolds (Huang et al., 2015; Jacob et al., 2017; Ghaedi et al., 2018; Deuse et al., 2019; de Carvalho et al., 2021). Importantly, the uniform repopulation of endothelial cells (re-endothelialization) is critical in reproducing functional vascular networks that play an essential role in gas exchange, barrier function, nutrient supply, and waste management in lungs (Calle et al., 2014). Gravity-driven and pump-driven seeding techniques have been used extensively to infuse endothelial cells into the scaffold (Scarritt et al., 2018). However, these methods have resulted in an inhomogeneous and incomplete distribution of cells, especially in the distal regions which leads to graft failure (Zhou et al., 2018). Further investigation and optimization of recellularization protocols are necessary to improve the functionality and sustainability of bioengineered lungs.

In silico models that employ computational fluid dynamics (CFD) are promising alternatives to costly experimental trial and error investigations, as they can serve as a virtually unlimited source of trials that can elucidate the mechanisms of cell seeding. Thus, using in silico models to define seeding parameters could ultimately lead to more efficient optimization strategies. For instance, Marín et al. used a CFD model to identify an optimal flow rate and perfusion pattern that provided the best cell seeding results in a commercially available synthetic scaffold with a regular porous microstructure (layers of cylindrical fibres of fixed diameter and distance apart) (Marín et al., 2018). Furthermore, they found that gravity and secondary flows were key factors in cell deposition. However, their simulations assumed the deposition of each cell upon contact with the scaffold surface and ignored cell adhesion factors such as biochemical composition of the scaffold (Chan and Leong, 2008), shear stress on the particle (Ott and Ballermann, 1995), and particle inertial impact (Darquenne, 2012; Olivares and Lacroix, 2012). As a result, the model presented by Marín et al. overestimated cell seeding efficiency by 35% compared to experiments. One example of deposition modelling within lung vasculature was done by Sohrabi et al. (Sohrabi et al., 2014) who aimed to quantify the transport and adhesion of drug particles. In their work, they used a particle-surface adhesion algorithm, herein referred to as the Decuzzi algorithm (Decuzzi and Ferrari, 2006). This algorithm predicted the probability of adhesion based on assumed particle-surface biochemical characteristics, particle shape and shear stress, and ultimately highlighted the importance of including particle adhesion mechanisms in the model. It should be noted that more recent works regarding particle adhesion in vasculature have been presented by the Decuzzi group, which model bonds between the wall and particle as linear springs that account for viscous effects (Coclite et al., 2017; Coclite et al., 2018; Coclite, 2020). However, these recent models were formulated using a Lattice Boltzmann method instead of traditional CFD methods, which iteratively solve for the Navier-Stokes equations. For the purposes of directly comparing cell deposition algorithms using traditional CFD methods, this study will be focusing on the Decuzzi formulation from 2006 (Decuzzi and Ferrari, 2006).

The contact algorithm and Decuzzi algorithm have been used throughout literature to describe biological deposition processes. However, particle inertial impact, which is critical for particles suspended in fluid with a diameter larger than 5 µm (Guha, 2008; Darquenne, 2012), is not accounted for in either of these algorithms. During sudden changes in fluid flow directions, the inertia of the particle causes it to continue along its initial trajectory. In reality this results in the deviation of the particle from the streamline and its eventual impact on the substrate. Considering the tortuous morphology of lung vasculature (Rahaghi et al., 2016), and the fact that human pulmonary endothelial cells have a diameter of 10–30 µm (Heath and Smith, 1979), an algorithm which accounts for particle inertial impact is critically needed yet not available in the literature.

Herein, a CFD model of mouse lung vasculature surrounded by acellular parenchyma was developed to understand and predict the effect of cell media flow rate on endothelial cell seeding. The model predicted the number and distribution of cells deposited by accounting for the particle inertial impact. Simulations were also performed with the implementation of the contact and Decuzzi algorithms of cell adhesion for comparison. Furthermore, experimental reseeding was performed on mouse lungs, and the histology results were compared with all three algorithms. This in silico tool aims to provide much needed insight to optimize recellularization strategies.

## Materials and Methods

### Reconstruction of Mouse Pulmonary Vasculature and Extracellular Matrix

A set of three-dimensional (3D) images of the lower right lobe of a 12-week-old adult C57BL/6 mouse lung injected with liquid contrast agent (Microfil, Flow Tech Inc. United States) was acquired using an X-ray micro-computed tomography system (SkyScan 1,172, Bruker Corporation) at a voxel resolution of 4.97 μm. To increase contrast and distinguish the vasculature from the void space in the images, a series of Gaussian smoothing (Gonzalez and Woods, 2018), and Hessian-based filtering (Jerman et al., 2016) was implemented. Afterwards, Otsu’s method (Otsu, 1979) was applied to binarize the image stack.

**FIGURE 1:**
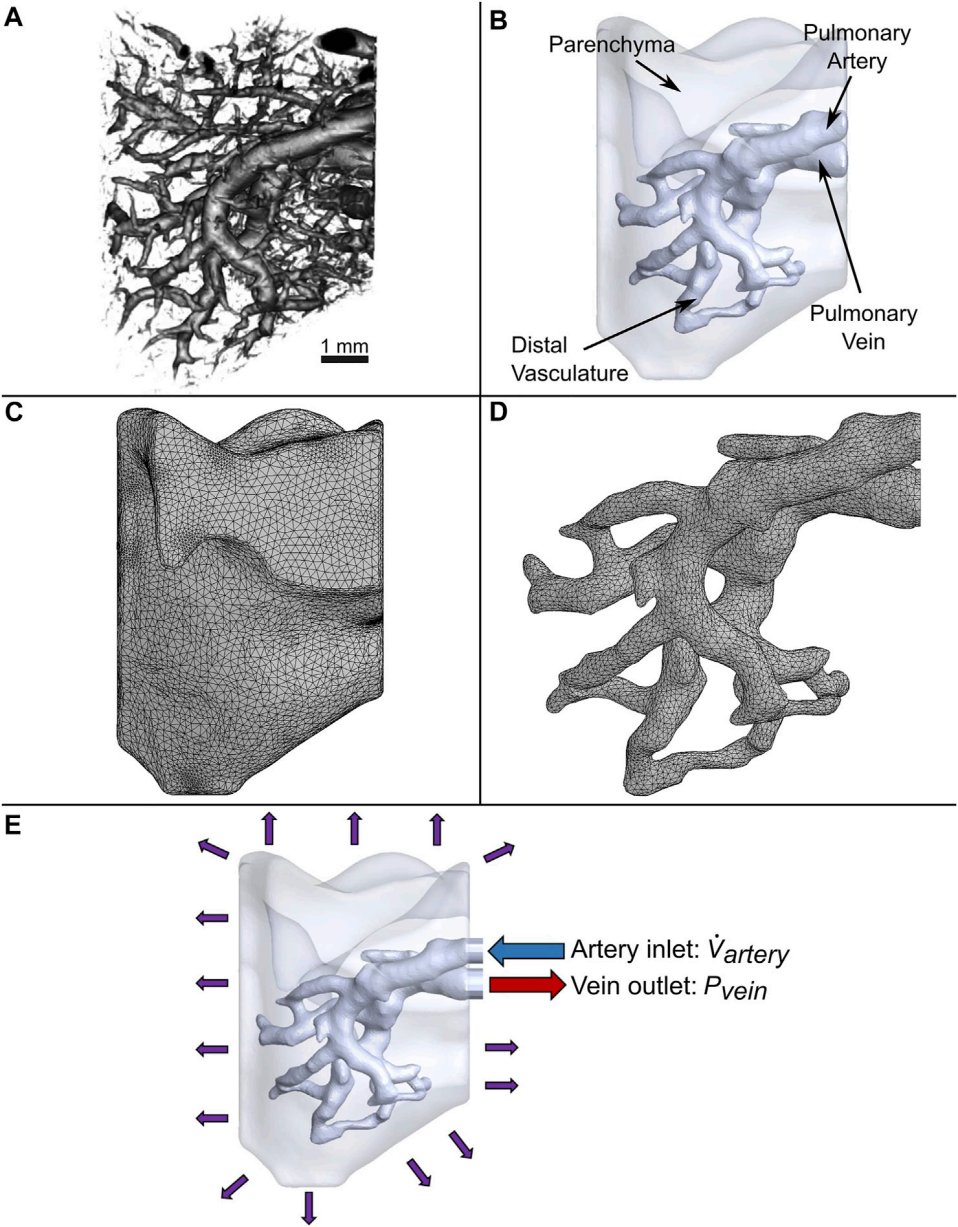
Generation of the computational domain for CFD simulations. **(A)** 3D micro-CT reconstruction of a native mouse lung. **(B)** Simplified model of vasculature surrounded by parenchyma modeled as a bulk uniform porous material. **(C)** Tetrahedral mesh of the parenchyma and **(D)** vasculature. **(E)** Applied boundary conditions.

The binarized images were imported into open-source medical image visualization software (3D Slicer (Fedorov et al., 2012)) for volume rendering of the vasculature, shown in Figure 1A. The lung vasculature and parenchyma were segmented separately to create two domains for the flow analysis, as shown in Figure 1B. Only a portion of the lung vasculature was delineated due to the high computational cost of modeling fluid and particle transport in such a complex vascular system. To do this, a threshold was set using the Segment Editor module of 3D Slicer so that only vessels of diameters greater than 200 microns were rendered. This diameter was found to be optimal for the subsequent operations. Next, any vessels that were isolated from the two largest groups of connected vasculature were subsequently removed. Connections were then manually created between branches of the two groups that were closest to each other. The final portion of the selected vasculature highlighted in Figure 1B consisted of an inlet section (defined here as the artery) that branched out into four generations of vessels (distal vasculature), which then consolidated into an outlet section (vein). The lung parenchyma was created by agglomerating all remaining vessels into a single porous body, also shown in Figure 1B.

### Fluid Domain Modeling

The vascular network and parenchyma geometry were imported into ANSYS (Release 2020 R1) to mesh the fluid volumes and perform transient modeling. All computing was conducted on a computer with 128 GB of memory and a processor with 16 cores and a base clock speed of 3.5 GHz. Element body sizing control was utilized to refine the vasculature mesh, since resolving the flow in this domain is critical for simulating the trajectory and deposition of cells. Furthermore, to accurately capture the boundary layer velocity gradient at the vessel walls, a fine mesh was created along these regions using inflation layer control. A total of 404,496 and 160,235 mixed elements for the parenchyma and vasculature were created, respectively, as shown in Figure 1C and Figure 1D. A mesh sensitivity analysis of the flow out of the vein was performed to ensure the accuracy of the simulation (Supplementary Figure 1). Only a 1.9% change in results was observed when doubling the number of elements.

It is assumed that the endothelial cells are bounded by the vessel walls (Scarritt et al., 2018). Consequently, the precise flow throughout the non-vascular parenchyma modelled by a detailed characterization of individual pores using a costly multi-million element mesh is beyond the scope of this work. Instead, the flow through the parenchyma domain was modeled as flow through a uniform porous material using Darcy’s law:

$$\nabla p=-\frac{\mu }{\alpha }u$$

where ∇p is the pressure gradient across the parenchyma [Pa], α is the permeability of the parenchyma [m], μ is the dynamic viscosity of the cell media [Pa s], and u is the velocity of the cell media [m s^−1^]. The permeability of the parenchyma was calibrated using experimental results from Engler et al. (Engler et al., 2019), who measured the distribution of flow through the vein and the rest of the lung at different perfusion rates, and was ultimately set as 8.9 × 10^–9^ cm^2^. This value falls well within the known range of 10^–12^–10^–8^ cm^2^ for Collagen I tissue (Tarbell and Shi, 2013), which the lung is largely comprised of (Laurent, 1986).

In our experiments, the decellularized lung scaffold is saturated with phosphate-buffered saline (PBS) before the cell media is injected (all experimental work is detailed in section 2.5). Therefore, multiphase modeling was used to characterize the interaction between the two fluids as the lung is recellularized. PBS and the cell media were represented as incompressible Newtonian fluids with densities of 1,005 kg/m^3^ (Brown et al., 2011) and 1,000 kg/m^3^ (Hinderliter et al., 2010), respectively, and dynamic viscosities of 1.02 cP and 0.94 cP (Fröhlich et al., 2013), respectively.

### Boundary Conditions

Flow rates for the re-endothelialization of mouse lung scaffolds have not been previously reported in literature. However, since the mechanical properties such as resistance and elastance of mouse and rat lungs are similar (Faffe et al., 2002), the re-endothelialization flow rate of rat lung studies were considered. These include the work of Peterson et al. (Petersen et al., 2010), who perfused rat lung scaffolds at 1–5 ml/min, and Scarritt et al. (Scarritt et al., 2018), who observed reseeding flow rates up to 20 ml/min.

To reflect this range of flow rates seen in literature for rat lungs, a peristaltic pump was used in our experiments (described in section 2.5.3) to inject the cell media at settings of 6 RPM and 15 RPM. These settings resulted in flow rates of 3.81 ml/min and 9.40 ml/min respectively, denoted as ${\dot{V}}_{low}$ and ${\dot{V}}_{high}$ respectively (listed in Table 1), and were applied to the model’s artery inlet.

**TABLE 1:** Cell seeding boundary conditions and values. Flow rate is specified at the inlet artery, and pressures are specified at the outlet vein.

Case	Arterial Inlet flow Rate [ml/min]	Maximum Reynolds Number	Venous Outlet Pressure [Pa]
${\dot{V}}_{\mathrm{low}}$	3.81	63	711
${\dot{V}}_{\mathrm{high}}$	9.40	157	2005

The lung scaffold was injected with 20 ml of cell media for each experimental trial. In preliminary simulations, it was noted that injecting approximately 0.2% of cell media was enough to fully saturate the vasculature. At this fully saturated point, the flow reaches steady state, and cell seeding efficiency begins to plateau (further elaborated on in section 3.2.1). Thus, simulating the injection of the first 1% of cell media was deemed to be sufficient for obtaining a final cell seeding efficiency value for each case. Simulating any further would not provide any new information and involve an unnecessary, costly increase to the computation time.

To represent the impedance throughout the vasculature caused by frictional losses and vessel wall compliance, a constant pressure boundary condition was applied to the vein outlet. This type of boundary condition is reasonable for simulations of steady and unsteady flows in a domain with a single vessel outlet (Grinberg and Karniadakis, 2008; Kheyfets et al., 2013). The values of pressure for the vein outlet were obtained from measurements of resistances throughout the vasculature and across the parenchyma of decellularized rat lung scaffolds at various flow rates in the work of Engler et al. (Engler et al., 2019). The pressure boundary condition values used in our work are listed in Table 1.

A porous jump condition was applied on the vessel wall to represent the porous nature of the acellular vasculature surfaces. This condition was used to model a thin membrane onto which the cells could deposit, while allowing the cell media to perfuse through. The permeability of this membrane was set to 8.9 × 10^–9^ cm^2^ to match that of the parenchyma.

### Cell Modeling and Deposition Probability

Mouse C166 endothelial cells (CRL2581, ATCC, Canada) were used in this study to repopulate the mouse lungs. These cells were modeled as a discrete phase of spherical particles with a diameter of 15 μm , which were measured with the Vi-CELL™ Cell Viability Analyzer (Beckman Coulter Life Sciences, US) and exhibited a density of 1,050 kg/m^3^ (Bryan et al., 2013). A cell concentration of 250,000 cells/ml was used for simulations to match the experiments.

The Lagrangian formulation was used to predict the trajectory of simulated particles caused by drag forces from the surrounding fluid, buoyancy, and gravity. This force balance equates the particle inertia with the forces acting on the particle, and is written as

$$m_{p}\frac{\text{d}u_{p}}{\text{d}t}=m_{p}\frac{\left(u-u_{p}\right)}{\tau_{r}}+m_{p}\frac{g\left(\rho_{p}-\rho \right)}{\rho_{p}}$$

**FIGURE 2:**
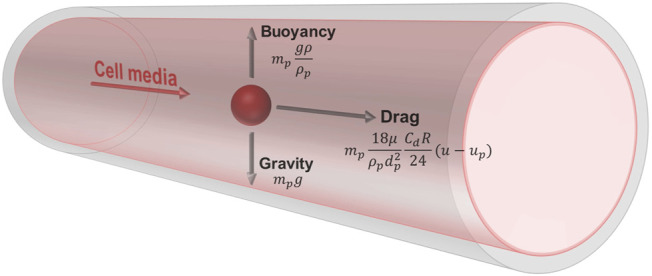
Free body diagram of a simulated cell suspended in media within the vessel. The cell has a density of 1,050 kg/m^3^ and diameter of 15 μm. The media has a density of 1,000 kg/m^3^ and viscosity of 0.94 cP. The trajectory of the particle is influenced by drag forces from the surrounding fluid, buoyancy, and gravity.

Where $m_{p}$ is the particle mass [kg], u ang $u_{p}$ are the fluid and particle velocity [m s^−1^], respectively, ρ and $\rho_{p}$ are the fluid and particle density [kg m^−3^], respectively, g is the gravitational constant [m/s^2^], and $\tau_{r}$ is the particle relaxation time [s]. $\tau_{r}$ characterizes the time period required for the particle to reach the free stream velocity (Gosman and Ioannides, 1983), and is given by

$$\tau_{r}=\frac{\rho_{p}d_{p}^{2}}{18\mu }\frac{24}{C_{d}Re}$$

where $d_{p}$ is the diameter of the particle [m], $C_{d}$ is the drag coefficient, and Re is the relative Reynolds number. Thus, Eq. (1) can be rewritten as

$$m_{p}\frac{\text{d}u_{p}}{\text{d}t}=m_{p}\frac{18\mu }{\rho_{p}d_{p}^{2}}\frac{C_{d}Re}{24}\left(u-u_{p}\right)+m_{p}\frac{g\left(\rho_{p}-\rho \right)}{\rho_{p}}$$

where the first term on the right-hand side represents drag force, and the second term represents buoyancy and gravity (Figure 2).

Since the cell phase accounted for less than 1% of the total volume in the medium, it was assumed that cell to cell interactions could be neglected, and that the presence of particles did not affect the flow of the cell media (Marín et al., 2018). Thus, one-way particle-fluid coupling was applied for simulations. The seeding or deposition of cells onto vessel wall surfaces was investigated using three unique particle deposition algorithms. Two of these algorithms, the contact algorithm and the Decuzzi algorithm, have been used throughout literature to describe biological deposition processes. A novel algorithm, herein referred to as the *Stokes algorithm*, was developed to simulate cell deposition more accurately. These are described in the following sections.

#### 3.4.1 Deposition Upon Contact Algorithm

The first deposition algorithm is herein referred to as the contact algorithm, which assumes that any particle that comes into contact with a wall’s surface is automatically deposited. Upon contact, the particle trajectory calculation is terminated. As a result, the algorithm does not take into account factors that affect cell adhesion and detachment such as biochemical composition (Chan and Leong, 2008), shear stress on the particle caused by the fluid (Ott and Ballermann, 1995), or particle impact velocity (Olivares and Lacroix, 2012).

#### 3.4.2 Decuzzi Cell Deposition Algorithm

The second cell deposition algorithm is the receptor-ligand algorithm developed by Decuzzi and Ferrari (Decuzzi and Ferrari, 2006), wherein the probability of cell deposition is expressed as a function of adhesive strength and dislodging forces. The adhesive strength of the cell depends on the stochastic binding of receptor molecules on the vessel walls and their counterpart ligand molecules on the cell surface. The dislodging forces are influenced by physiological factors such as cell shape and size, as well as shear stress caused by the media that flows around it. The Decuzzi deposition probability is defined as

$$P_{Dec}=m_{r}m_{l}K_{a}^{0}\pi r_{o}^{2}\exp\left[-\frac{\lambda d_{p}\mu S}{2k_{B}Tr_{0}^{2}m_{r}}\left[6\left(\frac{d_{p}}{2}+\delta_{eq}\right)F^{S}+2\frac{d_{p}^{2}}{r_{o}}T^{S}\right]\right]$$

where $m_{r}$ and $m_{l}$ are the surface densities of receptors and ligands [m^−2^], respectively, $K_{a}^{0}$ is the characteristic affinity constant of the ligand-receptor pair [m^2^], $r_{o}$ is the radius of the interaction surface [m], λ is the characteristic length of the ligand-receptor bond [m], μS is the shear stress [N m], $k_{B}T$ is the Boltzmann thermal energy [m^2^ kg s^−2^], $\delta_{eq}$ is the separation distance between the particle and surface [m], and $F^{S}$ and $T^{S}$ are the drag force and torque coefficients. Since the values of these parameters are not known for the decellularized lung, we applied the same values used in a study by Sohrabi et al. which investigated the deposition of drug particles in pulmonary vasculature (Sohrabi et al., 2014) (Table 2). With this algorithm, the deposition probability decreases with wall shear stress, which has been shown experimentally by Haun and Hammer (Haun and Hammer, 2008). However, like the contact algorithm, the particle impact velocity is not accounted for in this algorithm.

**TABLE 2:** Parameters used for the calculation of cell deposition probability using the Decuzzi algorithm (Sohrabi et al., 2014).

Parameter	Value
$m_{r}$	$10^{14}/{\text{m}}^{2}$
$m_{l}K_{a}^{0}$	$4.15\times 10^{-2}$
λ	$10^{-10}\;\text{m}$
$k_{B}T$	$4.14\times 10^{-21}\;{\text{m}}^{2}\text{kg}/{\text{s}}^{2}$
$\delta_{\mathrm{eq}}$	$5\times 10^{-9}\;\text{m}$
$F^{S}$	1.668
$T^{S}$	0.944

The Decuzzi cell deposition probability algorithm is executed as follows. Upon contact of the cell onto the vessel wall, the deposition probability is calculated for the cell, and a random number from a uniform distribution from 0 to 1 is generated. If the deposition probability is greater than the generated random number, the cell is deposited. Otherwise, the cell is reflected off the vessel wall surface to be carried further downstream by the media. The cell may impact the vessel wall again, where the algorithm is repeated, or ultimately exit the vasculature through the vein outlet.

#### 3.4.3 Stokes Cell Deposition Algorithm

Finally, we developed a novel cell deposition algorithm to account for the inertial impaction of the particle on the vessel wall surface. The inertia of the particle causes it to deviate from the streamline during sudden changes in flow and eventually impact the vessel wall. Thus, the greater the particle size and flow speed, the higher the probability for deposition on a surface. The Stokes number, which characterizes this behaviour of particles suspended in a fluid flow, is defined as

$$Stk=\frac{\rho_{p}d_{p}^{2}u}{18\mu d}$$

where $\rho_{p}$ and $d_{p}$ are the particle density and diameter, respectively, u and μ are the mean velocity and dynamic viscosity of the fluid, respectively, and d is the characteristic length equal to the diameter of the vessel. The probability of particle deposition on a substrate can be expressed as a function of the Stokes number (Darquenne, 2012). In this study, we devised an empirical cell deposition probability function which utilizes Stokes number as

$$P_{Stk}=\exp\left(-\frac{1}{Stk\cdot C}\right)$$

where C is an empirically adjusted inertia coefficient that accounts for the effect of Stokes number on deposition, as well as biochemical interactions and the truncated geometry of the vasculature. The higher the Stokes number and inertia coefficient, the more closely this probability function will approach 1, and the more likely that particles with be deposited. For low values of the Stokes number and inertia coefficient, this function will approach 0, meaning that particles will simply follow the streamlines of flow and have a lower chance of depositing onto the vessel walls. The Stokes cell deposition probability algorithm is executed in the same manner as the Decuzzi algorithm, where the cell is deposited if the calculated probability is greater than a randomly generated number.

#### 3.4.4 Quantification of Cell Deposition Uniformity

The uniformity of cell deposition is critical to the successful repopulation of the lung scaffold. To quantify this for the three algorithms, first the number of cells that were deposited on each facet of the vessel wall element mesh (Figure 1D) were counted. To determine how much this value varied throughout the facets of the vasculature, the statistical parameter known as the coefficient of variation was determined as

$$\text{Coefficient}\;\text{of}\;\text{Variation}=\frac{\sigma }{\mu }=\frac{\sqrt{\frac{\sum_{i=1}^{n}{\left(x_{i}-\mu \right)}^{2}}{n}}}{\mu }$$

where σ is the standard deviation of cell deposition, $x_{i}$ is the number of cells deposited on each facet of the vessel wall, μ is the mean of cells deposited per facet, and n is the total number of facets on the vessel wall, which was 20,872. A lower value of coefficient of variation demonstrates a more uniform distribution of deposited cells.

### Experimental Cell Seeding

#### 3.5.1 Lung Surgery and Decellularization

**FIGURE 3:**
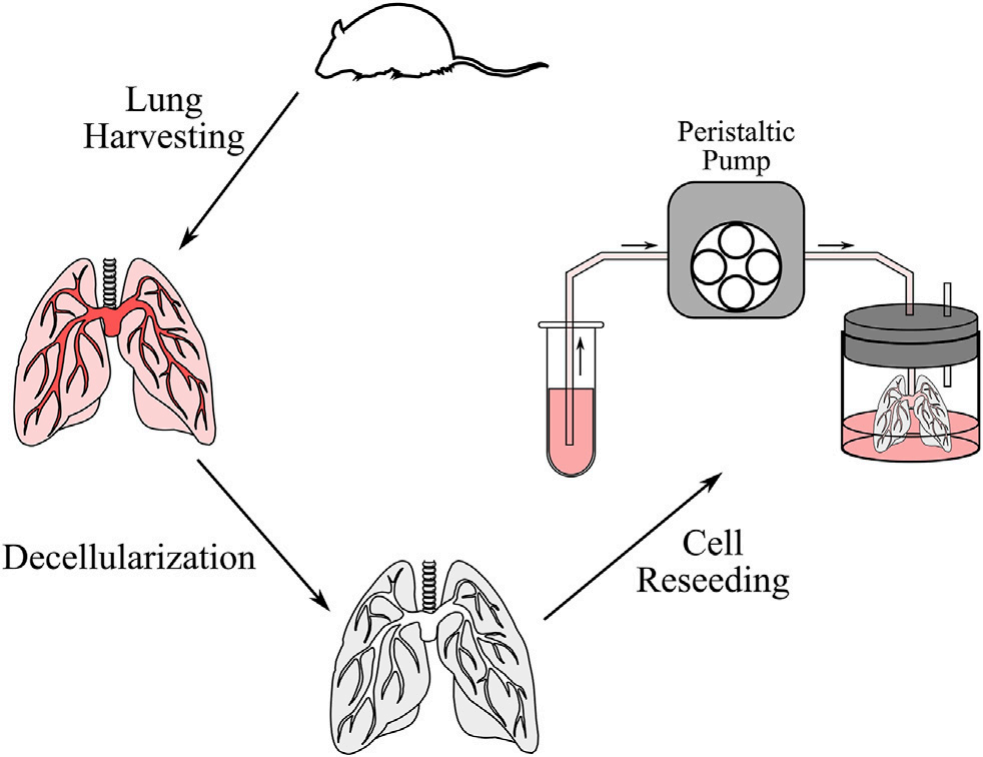
Schematic of the decellularization and cell reseeding process. After the lung was harvested from the mouse, the lung was perfused with decellularization solutions to remove cells and genetic content. The lung was then washed with distilled water and PBS, after which cells were reseeded through a peristaltic pump.

*Ex vivo* cell seeding was performed to validate the simulation results (Figure 3). Six lung scaffolds were generated from 12–14-week-old C57BL/6J strain male mice (Jackson Laboratory, United States). All animal studies were approved by the Institutional Animal Care and Use Committee of the University Health Network at the University of Toronto. After euthanasia, the mice were exsanguinated via injection of PBS (Thermo Fisher Scientific, CA) through the right ventricle. Following cannulation of the pulmonary artery and trachea, the heart-lung block was removed and stored in a solution of PBS and 1% antibiotic-antimycotic (Thermo Fisher Scientific, CA) at a temperature of 4°C until decellularization. The extracted sample can be stored for up to 1 week, but in this study, decellularization was performed immediately after.

Next, the decellularization procedure was completed as previously described by Daly et al. (Daly et al., 2012). In short, the samples were stored in distilled water at 4°C for 1 h, 0.1% Triton X100 solution at 4°C overnight, 2% sodium deoxycholate solution at 4°C for 24 h, 1 M sodium chloride solution at room temperature (∼25°C) for 1 h, and finally in 30 μg/ml porcine pancreatic deoxyribonuclease (Thermo Fisher Scientific, CA) solution at room temperature for 1 h. Between each solution, the airways and vasculature were rinsed with about 3 cc of distilled water. After decellularization, the heart-lung blocks were washed with PBS and stored with 1% antibiotic-antimycotic at 4°C for up to 2 days until reseeding was performed.

#### 3.5.2 Cell Culture

Mouse C166 endothelial cells (CRL2581, ATCC, Canada) were cultured in a solution containing 89% high glucose content Dulbecco’s Modified Eagle Medium (Thermo Fisher Scientific, CA), 10% Fetal Bovine Serum (Thermo Fisher Scientific, CA) and 1% antibiotic-antimycotic. The cells were stored in a standard incubator with an environment of 95% air and 5% CO_2_ at 37°C. The cell culture media was changed every 2–3 days. Finally, the cells were trypsinized and then counted using the Vi-CELL™ Cell Viability Analyzer (Beckman Coulter Life Sciences, US). Five million mouse C166 cells were suspended in 20 ml of cell culture for reseeding, resulting in a cell concentration of 250,000 cells/ml.

#### 3.5.3 Cell Reseeding

The cell suspension reservoir was placed at the same height as the bioreactor containing the decellularized lung to avoid any effects due to gravitational pressure head. Flexible tubing (ID = 3.1 mm; Cole Parmer, CA) was used to connect the cell suspension to the cannulated pulmonary artery. Perfusion seeding was completed using a Masterflex Laboratory Standard (L/S). Cells were delivered using pump settings of 6 RPM and 15 RPM to achieve average flow rates of 3.81 ml/min and 9.40 ml/min for three samples each. After seeding, a 1 ml sample of post-perfusion cell media was collected to estimate the cell seeding efficiency. The samples were allowed 18 h of static culture to facilitate initial adhesion.

#### 3.5.4 Histological Analysis

To fix the samples, a 10% formalin solution was first injected intratracheally and stored at room temperature overnight. Next, the samples were transferred into a 70% ethanol solution to remove water content. Then, the samples were processed using he Excelsior ES Tissue Processor (Thermo Fisher Scientific, CA), where an intermediate solvent xylene displaced the ethanol solution and removed fat from the tissue which otherwise presented a barrier to wax infiltration. After this step, the samples were embedded into paraffin blocks. Three 5 μm thick whole lung sections from each lung were stained by hematoxylin and eosin (H&E). H&E slides were prepared according to protocols established by Wallis et al. (Wallis et al., 2012). The slides were scanned using the Aperio slide scanner (Leica Biosystems, United States). Finally, the images were processed with HALO™ software (Indica labs, United States) to quantify the amount of cell coverage. Here, the surface area of the stained cells and the uncovered scaffold were quantified. The cell surface coverage percentage could then be calculated, defined as

$$CSC\%=\frac{\text{Cell}\;\text{Surface}\;\text{Area}}{\text{Cell}\;\text{Surface}\;\text{Area}+\text{Uncovered}\;\text{Scaffold}\;\text{Area}}\times 100\%$$

## Results

### Model Flow of the Cell Media

**FIGURE 4:**
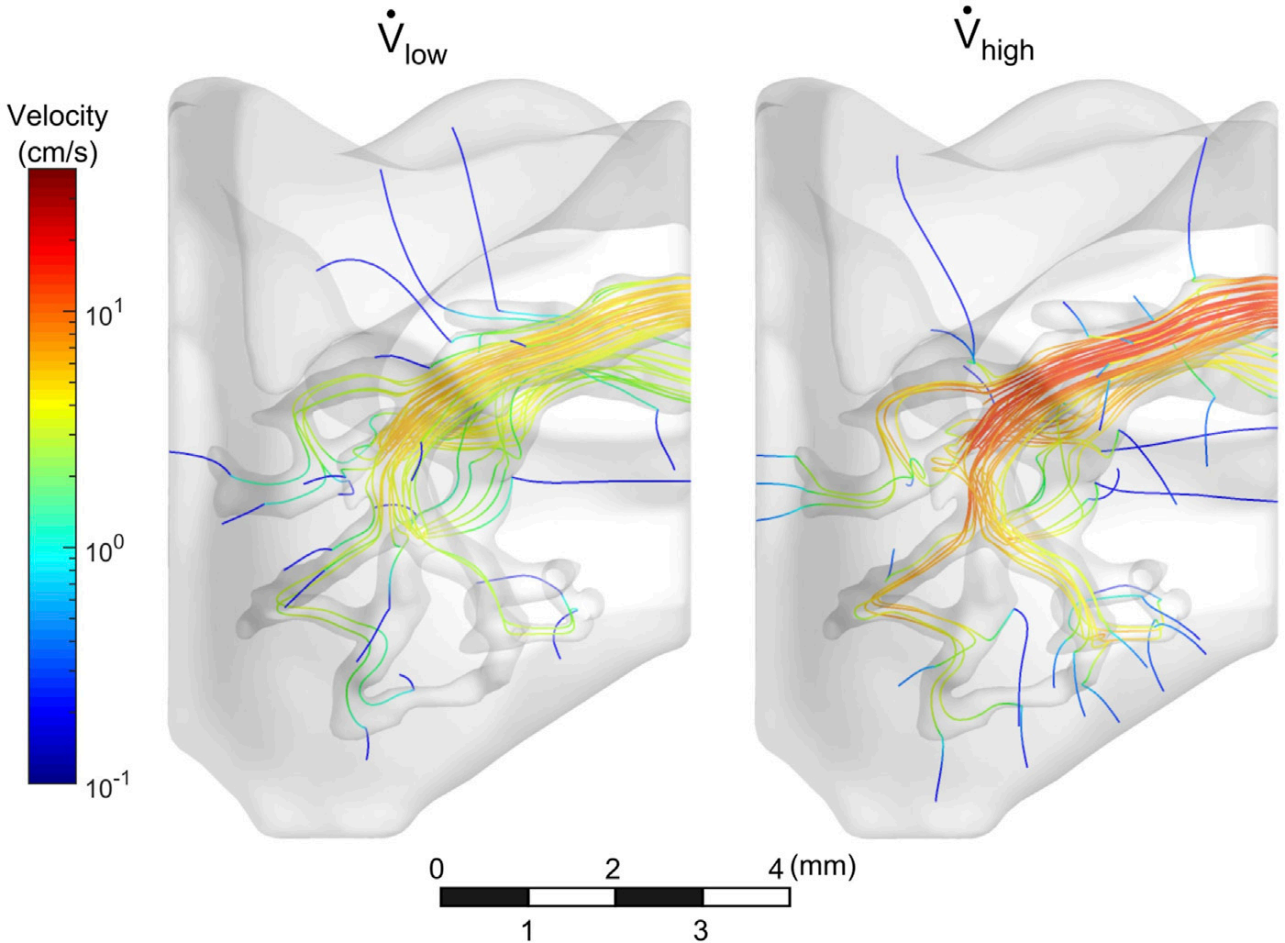
Velocity streamlines throughout vasculature. Streamlines circulated out the PV, while some permeated through vessel walls.

The flow field of the mouse lung vasculature was visualized using streamlines which originate from the PA inlet, as shown in Figure 4. These lines represent the direction in which the cell media traveled during steady-state flow. For both the low and high flow rate cases, as the fluid circulated throughout the vasculature, a portion permeated through the porous vessel walls, while the rest exited through the PV outlet. In general, streamlines permeated out of the vasculature in regions of high tortuosity such as the distal vasculature. For the high flow rate case, more streamlines were seen in the distal regions than compared to the low flow rate case. Furthermore, more fluid exited the domain through the PV in the low flow rate case. The percentage of flow through the PV was 34 and 25% for the low and high flow rate cases, respectively.

**FIGURE 5:**
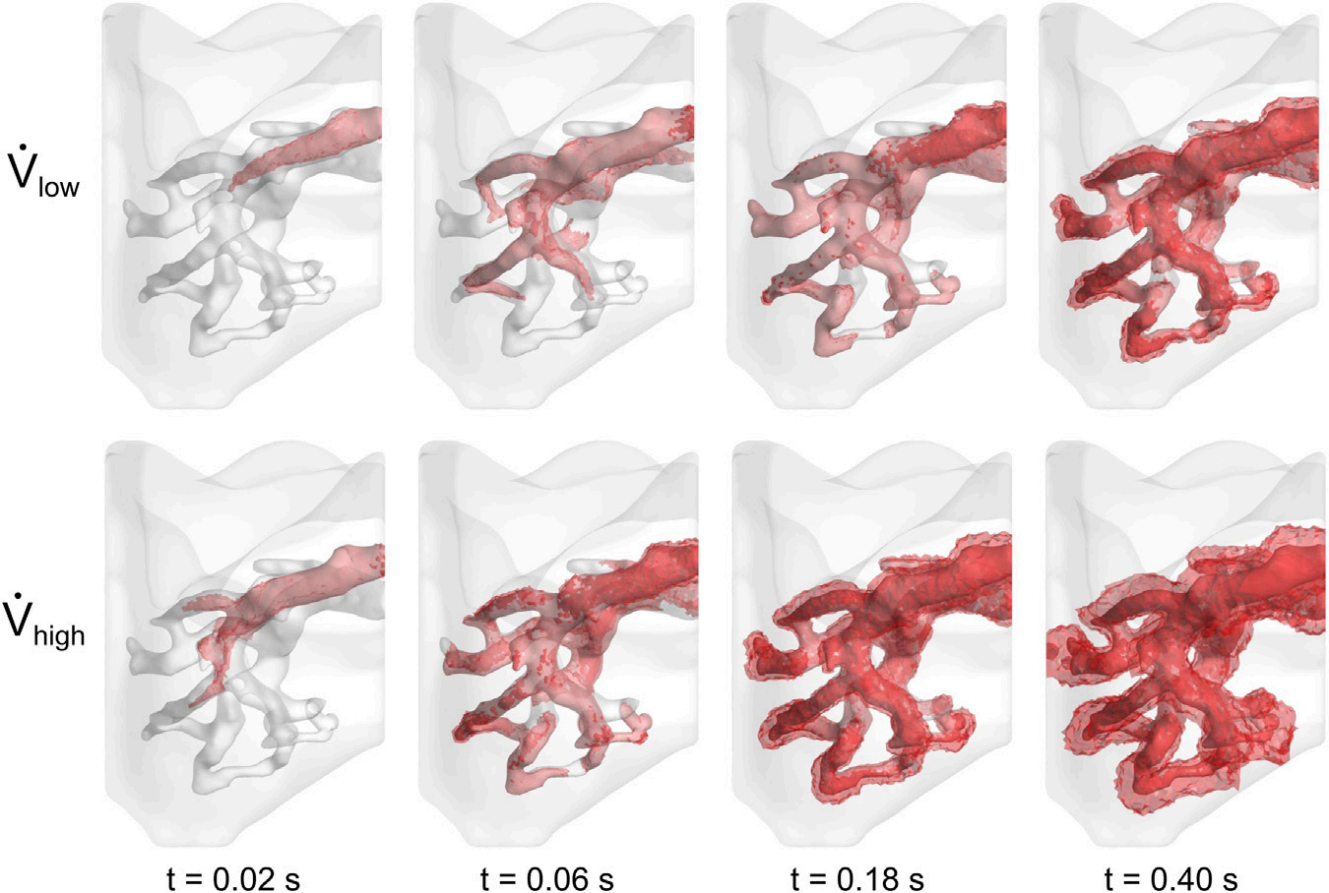
Perfusion of cell media throughout lung vasculature. Cell media permeated through the vessel walls at 0.50 and 0.25 s for the low and high flow rate cases, respectively.

The cell media flow was also visualized in Figure 5. In both cases, the media preferentially egressed through the PV before it reached all distal regions of the vasculature. Approximately 0.5 s were required for the cell media to fully perfuse throughout the vasculature for the low flow rate case, whereas it took less than 0.25 s for the high flow rate case. For both cases, as time progressed, the cell media permeated through the vessel walls and into the surrounding parenchyma.

### Cell Deposition on the Vessel Wall

**FIGURE 6:**
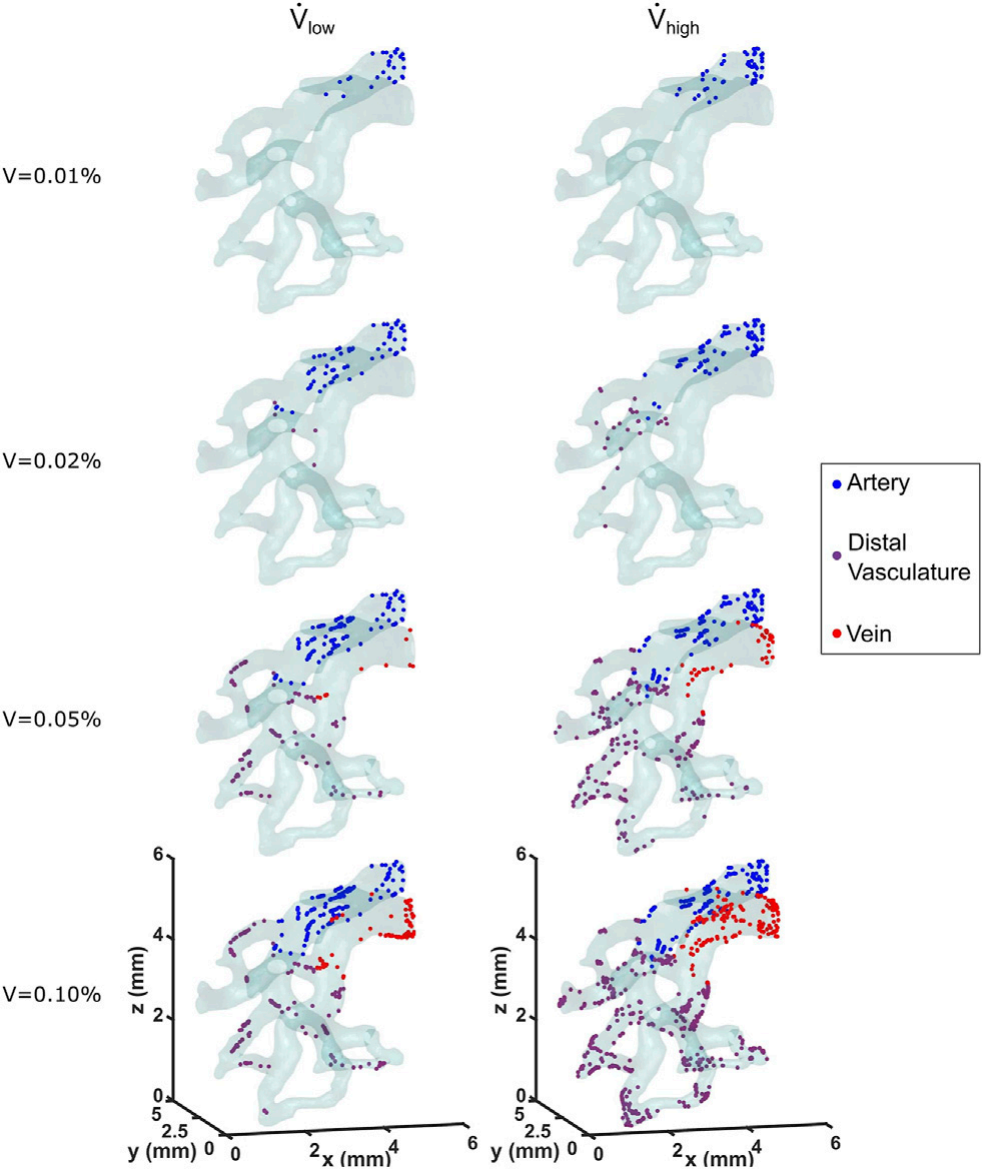
Locations of deposited cells are shown as a function of cell media injected for the low and high flow rate cases. The Stokes cell deposition algorithm was used here, with an inertia coefficient of *C* = 300.

The contact, Decuzzi, and Stokes cell deposition algorithms were used to simulate cell seeding for the low and high flow rate cases. The locations of seeded cells for both cases using the Stokes algorithm with *C* = 300 is compared in Figure 6. Cell deposition began along and remained limited to the arterial wall for the first 0.01% of cell media injected. By the time 0.2% of volume was injected, 22 cells were deposited along the distal vasculature for the high flow rate case, whereas only 5 were deposited in the region for the low flow rate case. The cells continued to deposit throughout the vessel wall as the amount of volume injected increased. At the moment where 0.1% of the cell media was injected, the high flow rate case exhibited 64.5% more cells deposited compared to the low flow rate case. Moreover, they appeared to be more uniformly distributed throughout the different sections of the vasculature for the high flow rate case as well.

#### 4.2.1 Comparison of Cell Seeding Efficiency

**FIGURE 7:**
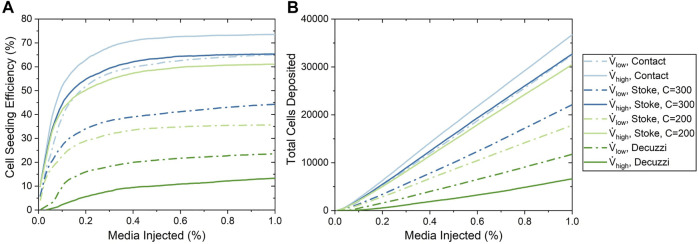
Cell seeding efficiency and the total number of cells deposited as a function of the percentage of media injected for various cell seeding algorithms. **(A)** Cell seeding efficiency. **(B)** Total number of cells deposited.

Cell seeding efficiency and the total amount of cells deposited as a function of percentage of cell media injected using the three deposition algorithms are shown in Figure 7. For all of these deposition algorithms, cell seeding efficiency began to plateau when approximately 0.2% media was injected, which resulted in a linear increase of the total number of cells deposited. This milestone corresponded to when the cell media had first perfused through the entire vasculature, thus establishing the maximum available surface area for the cell deposition. The contact algorithm predicted the highest cell seeding efficiencies of 65.1 and 73.5% for the low and high flow rate cases, respectively.

**FIGURE 8:**
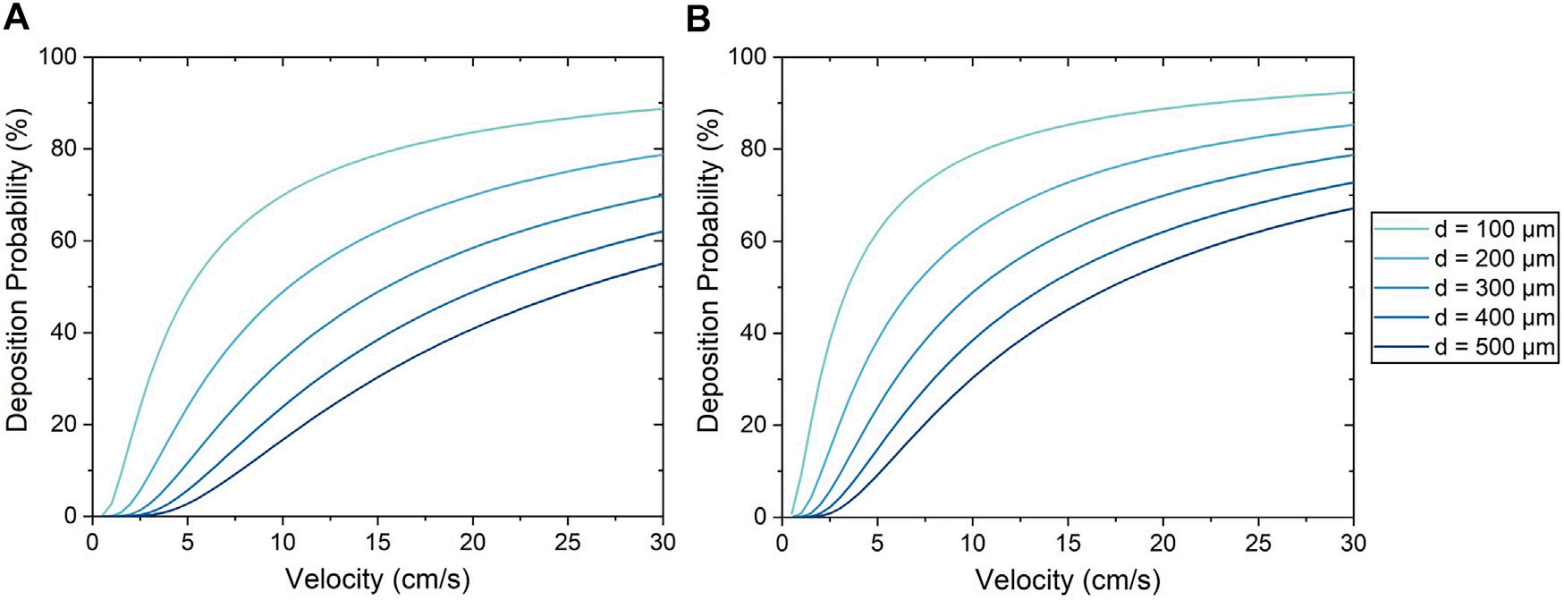
Cell deposition probability using the Stokes algorithm with an inertia coefficient of **(A)** *C* = 200 and **(B)** *C* = 300.

For the Stokes algorithm, the inertia coefficient of *C* = 300 led to the best agreement with experiment results. The Stokes algorithm with *C* = 200 is also presented here to demonstrate the effects of changing the inertia coefficient. The deposition probabilities for different velocities and vessel diameters using these two inertia coefficients are shown in Figure 8. For example, a cell impacting a vessel wall with a diameter of 200 μm at 5 cm/s had a deposition probability of 23.9% for *C* = 200 and 38.5% for *C* = 300. Cell deposition probability increased with increasing velocity and decreased with increasing vessel diameter. This was due to the emphasis on particle inertial impact in the Stokes algorithm; the high velocity and small vessel diameter resulted in the tendency for particles to deviate from the flow streamline and deposit onto the vessel wall. Referring back to Figure 7, The seeding efficiencies using *C* = 300 were 44.3 and 65.3% for the low and high flow rates, respectively, while they were 35.7 and 61.1% using *C* = 200.

Using the Decuzzi algorithm, the cell seeding efficiencies were the lowest at 23.5 and 13.3% for the low and high flow rates, respectively. Unlike the contact and Stokes algorithms, the Decuzzi algorithm predicted that a higher flow rate resulted in fewer cells deposited. The difference between the Decuzzi algorithm and the others is attributed to how it accounts for particle dislodging forces due to higher shear stress levels present at higher flow rates.

#### 4.2.2 Cell Seeding Distribution and Uniformity

**FIGURE 9:**
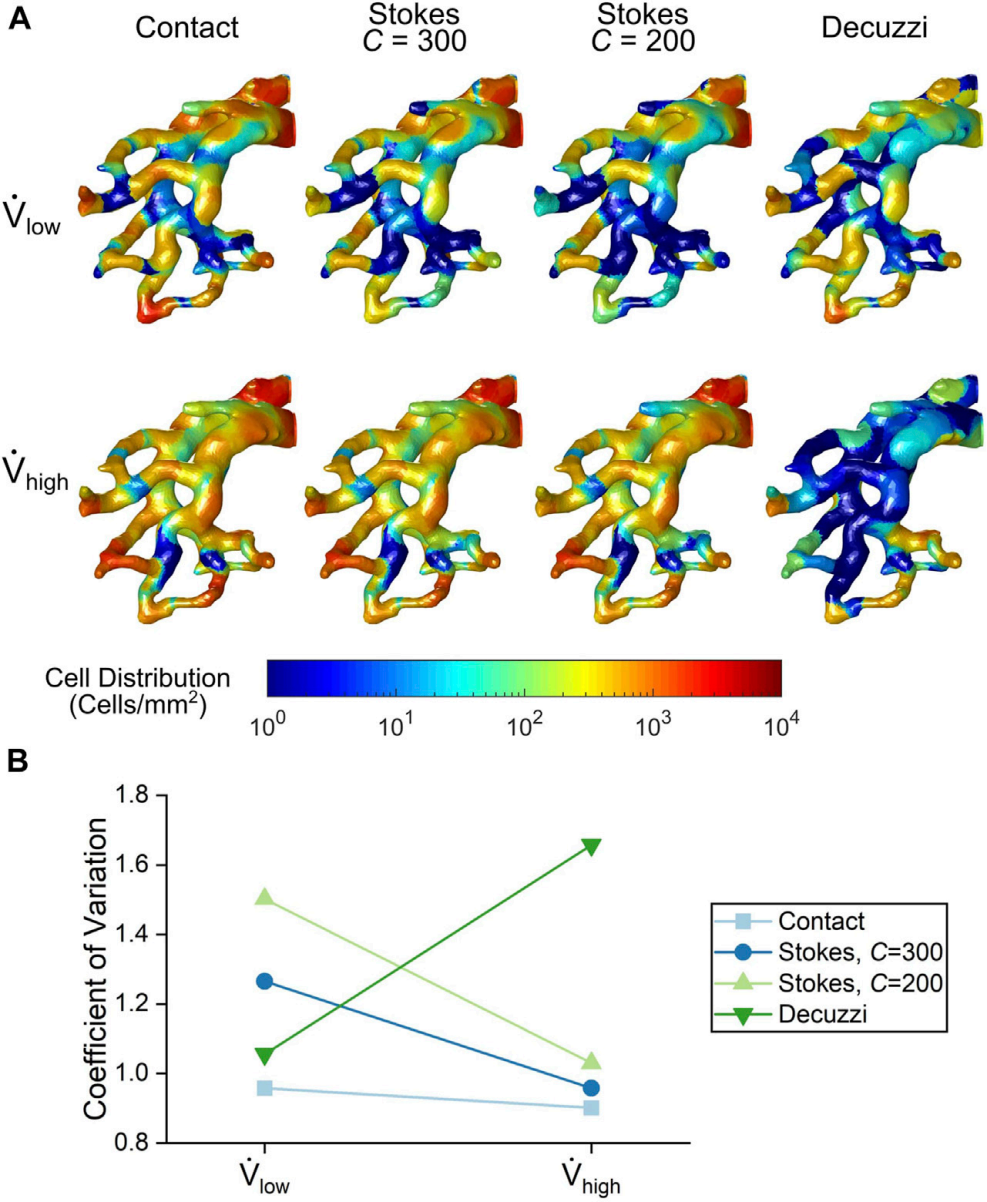
**(A)** Cell seeding distribution contours from each cell seeding algorithm. **(B)** Variance quantification of cell distribution uniformity for each cell seeding algorithm.

A contour map of seeded cells on the vessel walls is shown in Figure 9A. In general, large populations of cells were seen near the PA inlet and regions of high tortuosity. These regions had streamlines that rapidly change direction (see Figure 4) and therefore the particles were more likely to contact the vessel wall. Conversely, regions with wide, long, and straight segments had lower cell deposition.

For the contact and Stokes algorithms, the number of seeded cells increased with the higher flow rate. The uniformity of seeded cells also increased for these algorithms, as seen by a more even colour distribution at the high flow rate. This trend was quantified using the coefficient of variation of deposited cells shown in Figure 9B. For the contact and Stokes algorithms, the variance decreased with flow rate, demonstrating that uniformity increased. In contrast, for the Decuzzi algorithm, both the number and uniformity of deposited cells decreased for the high flow rate case, as illustrated by the rise in the variance of deposited cells.

### Experimental Validation of Simulations

*Ex vivo* reseeding of endothelial cells was performed on six mouse lung scaffolds; three at the low flow rate (average of 3.81 ml/min) and three at the high flow rate (average of 9.40 ml/min). During the experiments, it was noted that the lung expanded while cell media was injected into the vasculature. This change in geometry was not taken into consideration in the model, as a rigid body was assumed in the simulations. The results for experiments are presented as the mean ± standard error when applicable.

**FIGURE 10:**
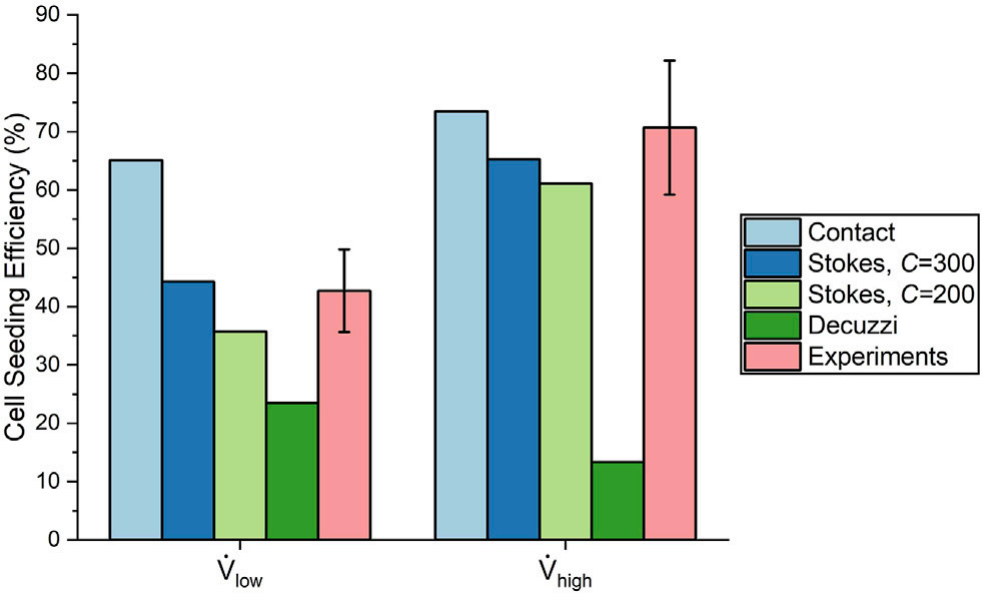
Comparison of cell seeding efficiency from each cell deposition algorithm and experiments.

The efficiencies from the experiments were 42.7 ± 7.1% and 70.7 ± 11.5% for the low and high flow rate cases, respectively. This represents a 66% increase in seeding efficiency by using the high flow rate. These experimental values are compared to the final seeding efficiencies of the algorithms in Figure 10. The contact algorithm overestimated cell deposition, especially for the low flow rate case, where it had overpredicted the efficiency by 22.4%. The Stokes algorithm with *C* = 300 matched experimental results well, with percentage errors of 3.7 and 7.6% for the low and high flow rate cases, respectively. The Stokes algorithm with *C* = 200 slightly underestimated cell deposition, with percentage errors of 16.4 and 13.6% for the low and high flow rate cases, respectively. Meanwhile, the Decuzzi algorithm severely underestimated cell deposition, especially for the high flow rate case where it underpredicted seeding efficiency by 57.4%. When comparing the Stokes algorithm with *C* = 300 and the Decuzzi algorithm for the high flow rate case, we observe that the percent error decreased by 73.9% when using the Stokes algorithm.

**FIGURE 11:**
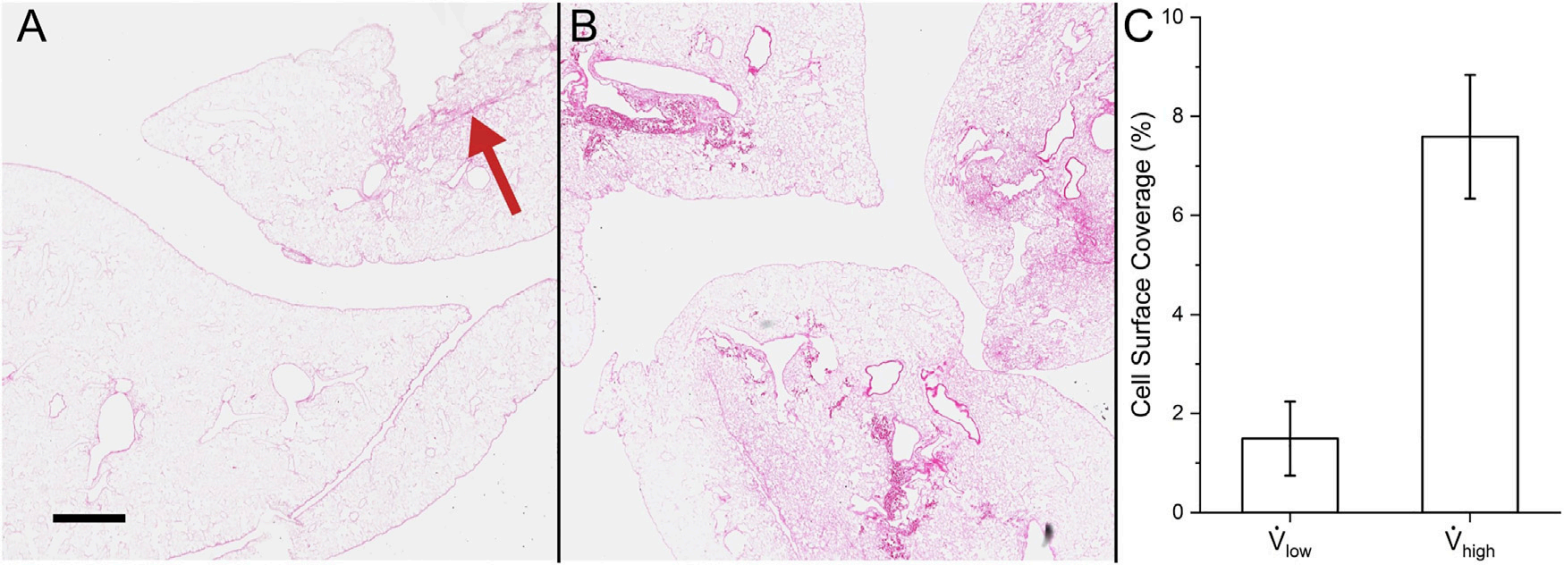
Cell surface coverage in experiments. Hematoxylin and eosin stain of **(A)** reseeding with low flow rate (red arrow indicates the small group of cells) and **(B)** high flow rate. **(C)** Quantification of the lung surface area that is seeded. Scale bar is 500 *µ*m.

The histology and cell surface coverage results are shown in Figure 11. Deposited cells were sparse for lungs seeded at the low flow rate. Only one small group of cells can be seen in the top right of Figure 11A. On the other hand, many more cells were deposited for the lungs seeded at the high flow rate (Figure 11B). Moreover, the cells were distributed more uniformly using the high flow rate compared to the low flow rate. The cell surface coverage was 1.49 ± 0.75% and 7.59 ± 1.25% for the low and high flow rates, respectively. This trend of higher deposition and greater uniformity at the high flow rate matches the results from the contact and Stokes algorithms.

## Discussion

This study presented a computational fluid dynamics model capable of predicting transient cell deposition for a variety of fluid flow conditions in an acellular mouse lung scaffold. Furthermore, a novel Stokes cell deposition algorithm was implemented and compared to the contact and Decuzzi algorithms to elucidate the most accurate representation of the mechanism underlying cell seeding. It was shown that the contact algorithm overestimated cell deposition by up to 22%. This overestimation was expected, given the automatic deposition of cells does not account for any adhesion mechanics (Marín et al., 2018). On the other hand, the Decuzzi algorithm severely underestimated cell deposition, especially for the high flow rate. This difference may be partly attributed to the fact that the exact values of the parameters of the Decuzzi algorithm for the decellularized lung are not known. More importantly, the Decuzzi algorithm uses an inverse relationship between deposition probability and shear stress, which predicts low cell deposition at high flow rates where shear stress is high. On the other hand, the Stokes algorithm, which accounts for inertial impact, demonstrated strong agreement in predicting cell deposition. Although high shear stress does indeed lead to the detachment of cells (Rosenman et al., 1985; Feugier et al., 2005), it was identified experimentally that the higher flow rate led to enhanced cell deposition. From these observations, it follows that for the flow rates studied here, cell deposition is influenced less by shear stress, and more by the inertial impact of the cell. This phenomenon was also observed by Melchels et al. (Melchels et al., 2011), who reported greater cell densities in regions of the scaffold with higher flow rate and shear stress.

Moreover, it was observed from experiments that the uniformity of cell deposition increased with increasing flow rate. The Stokes algorithm was also able to predict this trend in cell deposition uniformity, and helps to make sense of this relationship; as shown in Figure 8, the Stokes deposition probability varies significantly between vessels of different diameters at low flow rates (e.g. 11–64% at 5 cm/s with *C* = 300). However, at higher flow rates, the disparity of deposition probability between vessels of different diameters is decreased (e.g. 64–91% at 25 cm/s with *C* = 300), resulting in increased uniformity. This agreement with experimental results further demonstrates the capability of the Stokes algorithm to accurately model cell deposition.

A good agreement between in silico results with the Stokes deposition algorithm and *ex vivo* cell seeding was found. By accounting for the inertial impact of the cells on the vessel walls, the Stokes algorithm accurately predicted increased cell deposition and increased uniformity with increasing flow rate. The contact algorithm consistently overestimated cell seeding due to its assumption of automatic particle deposition, whereas the Decuzzi algorithm consistently underestimated cell seeding due to its overemphasis on particle dislodgement forces caused by fluid shear stress. However, only two flow rates were explored in this study. The extent to which the trend of cell deposition can increase with flow rate must be further explored. Higher velocities may generate critical shear stresses which could result in mass cell detachment or cell death (Tang et al., 2012). This result was observed by Baptista et al. (Baptista et al., 2016), who noted that reseeding liver scaffolds at 40 ml/min resulted in significantly lower cell proliferation and higher apoptosis and cytotoxicity. In this study’s formulation of the Stokes algorithm, the probability of deposition is solely dependent on the Stokes number and does not account for a critical flow rate or shear stress. A deposition algorithm that incorporates both the shear stress mechanics described by the Decuzzi formulation and the inertial impact mechanism described by the Stokes formulation could significantly increase the range of flow rates that this tool is valid for. Additionally, as research into the effect of biochemical interactions advances, implementation of these effects could further increase this algorithm’s accuracy.

Furthermore, it is important to acknowledge that the model was created based on a single lobe of the mouse lung and with truncated vasculature due to computational costs. Although the fluid resistance of the full lung vasculature was represented by implementing a pressure value at the outlet, a significant portion of vasculature surface area was omitted in this study. This omission could affect the model’s cell seeding efficiency, as the occurrence of cells contacting the vessel wall increases with the surface area of the wall and therefore have more chances to deposit. Moreover, the Stokes number, and therefore cell deposition, depends largely on fluid velocity and diameter of the vessel. Since the morphology of lung vasculature varies throughout the lung (Huang et al., 1996), the effect of incorporating more vessels into the model should be investigated.

Initial cell deposition is critical for the success of recellularized organs (Holy et al., 2000; Saini and Wick, 2003). By creating a model which accurately simulates cell seeding by accounting for the particle’s inertial impact, flow rate was found to be an important parameter for increasing seeding efficiency. The CFD tool presented in this study holds great potential in helping researchers analyze how bioreactor parameters can affect cell seeding in the lung scaffold, all while saving time and costs associated with experimental trials. Conditions which maximize cell seeding may be further elucidated through computational analysis, ultimately optimizing the lung recellularization process.

## Data Availability

The raw data supporting the conclusions of this article will be made available by the authors, without undue reservation.
