# Supplementary material for: Cell Inertia: Predicting Cell Distributions in Lung Vasculature to Optimize Re-endothelialization
Source: Front Bioeng Biotechnol. 2022 Apr 27;10:891407. doi: 10.3389/fbioe.2022.891407 (PMC9092599; doi:10.3389/fbioe.2022.891407)
Supplement: Supplementary file 1 [file DataSheet1.docx]

**Supplementary Material**

A mesh dependency study was completed to verify the accuracy of the results with respect to mesh size. Simulations were performed with different mesh sizes using the boundary conditions corresponding to the $\dot{V}_{high}$ case, and the percent of flow exiting the vein was compared (Supplementary Figure 1). When the element count was approximately doubled from 271,996 to 564,731, outflow through the vein changed from 25.9% to 25.4% - a percent difference of only 1.9%. This investigation showed that at a total element count of 564,731, the fluid field results are independent of element size. Since particle movement is entirely dependent on the fluid field, the resulting number of particles deposited should also be independent of element size.


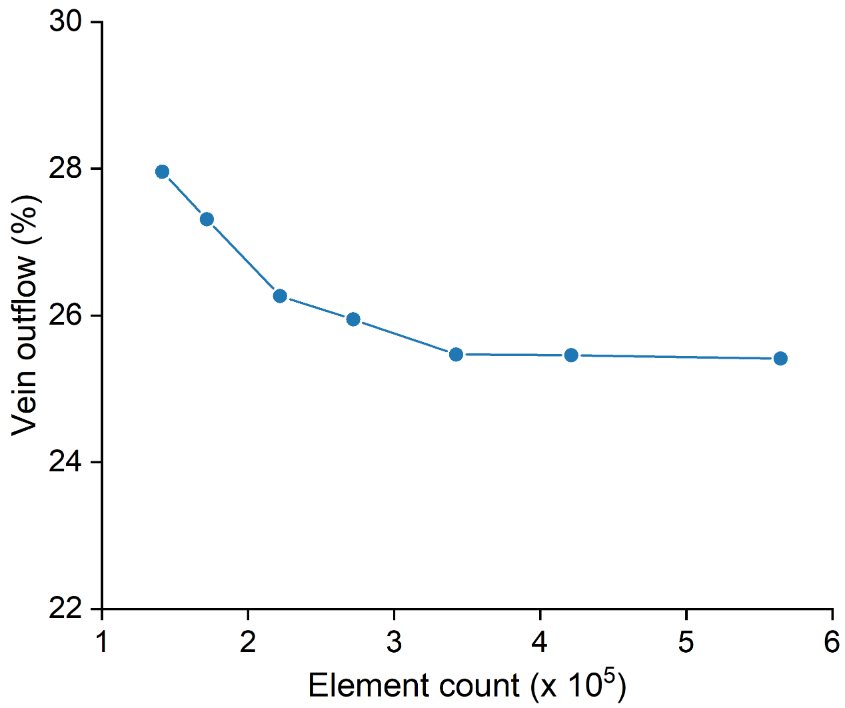


**Supplementary Figure 1**. Percent of cell media that flowed out through the vein as a function of mesh elements.
